# Bridging the Clinic to Community: Music Performance as Social Transformation for Military Service Members

**DOI:** 10.3389/fpsyg.2019.00119

**Published:** 2019-02-05

**Authors:** Rebecca Vaudreuil, Hannah Bronson, Joke Bradt

**Affiliations:** ^1^National Endowment for the Arts, Washington, DC, United States; ^2^National Intrepid Center of Excellence, Bethesda, MD, United States; ^3^Department of Creative Arts Therapies, Drexel University, Philadelphia, PA, United States

**Keywords:** performance, music, music therapy, social transformation, military service members, traumatic barin injury, post traumatic stress disorder

## Abstract

The use of music performance in music therapy with military service members is discussed as a vehicle for social transformation and reintegration. The use of performance in music therapy is not without controversy primarily because therapy is considered a process, not a product, and confidentiality and privacy are essential components of therapy. However, others have argued that public performances can validate therapeutic changes in clients, give voice to their experiences, raise awareness of social issues within their communities, transform perceptions of injury, or illness in audience members, and may result in the clients gaining support and validation from their communities. We discuss the potential of music performances to contribute to individual development, reinforce rehabilitation, enhance function, and facilitate change at the community level to support reintegration of military service members. We illustrate this through two brief case reports of service members who received music therapy as part of their treatment for post-traumatic stress disorder, traumatic brain injury, and other psychological health concerns at the National Intrepid Center of Excellence, a Directorate of the Walter Reed National Military Medical Center, Bethesda, MD, United States. The service members wrote, learned, and refined songs over multiple music therapy sessions and created song introductions to share with audiences the meanings and benefits gained from integrating performance in music therapy. The case reports also include excerpts of interviews conducted with these service members several months after treatment about their experiences of performing and the perceived impact of their performances on the audience and greater community.

## Introduction

Music performance is an integral part of the creative process. Whereas the general public may associate the concept of performance with entertainment, performances are increasingly used to stimulate discourse related to socio-political and psychological issues (e.g., Yotis et al., 2017). In music therapy, performance is utilized to give voice to clients’ experiences and facilitate greater understanding of their struggles Baker, 2013; O’Grady et al., 2015). Recently, media outlets have reported on music performances by military personnel who have sustained physical and/or psychological injuries (Public Broadcasting Service, 2017; KXTV, 2018). Little attention has been paid in the literature, however, to the unique benefits and challenges of the use of performance with military populations. The purpose of this perspective article is to discuss the potential for growth and social transformation for service members when integrating performance into music therapy. In addition, we present considerations to ensure that performance is an enriching experience for service members and audiences. We illustrate this through two brief case reports of service members who chose to integrate performance into music therapy treatment.

## The Use of Performance in Music Therapy

Board-certified music therapists use a variety of music experiences within a therapeutic relationship to address individualized goal areas (e.g., emotional, cognitive, physical, social, spiritual) (American Music Therapy Association [AMTA], 1998–2018), which may include music-guided relaxation, expressive/(e.g., singing, instrument playing) and receptive/(e.g., listening to live/recorded music) music engagement, and songwriting. The focus of music therapy is on the therapeutic process, not the musical product (Ansdell, 2010); therefore, many music therapists are opposed to public performances by clients as this may blur boundaries of privacy and confidentiality (Ansdell, 2010). Additionally, music therapists have cautioned against incorporating performance because performance-related stress and potential adverse audience responses may put clients at risk (Turry, 2005). However, music therapists who practice within a community music therapy model (Ansdell, 2005; Soshensky, 2011; Stige and Aarø, 2012) have proposed that performance has significant potential for enhancing clients’ health and psychosocial well-being. Ruud (2004) argued that performances in music therapy facilitate negotiation of “space between the private and the public, the client and the institution, or the client and the community.” O’Grady et al. (2015) encourage music therapists to “shift the focus to *when*, not *if*, performance might be considered an appropriate method in music therapy” (p.124).

Performance integrated in music therapy offers opportunities for personal transformation and social change. First, performance provides a context for clients to creatively connect with audiences and share their experiences through artistic expression (Day et al., 2009). Through performance, clients can bring their experiences into a public space, which can raise awareness about personal struggles and societal (mis)perceptions, including mental health stigma (O’Grady, 2009). Second, it provides opportunities for clients to step outside of their comfort zones through structured risk-taking (Baker, 2013). Third, performance allows clients to demonstrate their skills and take pride in creativity (Baker, 2013). Fourth, positive feedback and public acknowledgment may enhance a client’s self-confidence and motivation (O’Grady, 2009; Baker, 2013). Finally, performing can reciprocally impact the client-audience relationship: audiences can witness healthy parts of clients rather than deficits, and clients can feel supported by audiences, potentially resulting in transformative experiences (Soshensky, 2011; Baker, 2013).

## Music Performance by Military Service Members

### Music Therapy in Military Settings

Music therapy is “a reflexive process wherein the therapist helps the client to optimize the client’s health, using various facets of music experience and the relationships formed through them as the impetus for change” (Bruscia, 2014, Chapter 4, para. 5). Music therapy is utilized in the United States to treat service members recovering from service-related injuries, specifically traumatic brain injury (TBI) and posttraumatic stress disorder (PTSD). The National Endowment for the Arts (NEA) Creative Forces: NEA Military Healing Arts Network is a partnership with the United States. Departments of Defense and Veterans Affairs and the state and local arts agencies with administrative support provided by Americans for the Arts. Creative Forces programming places creative arts therapies at the core of treatment models in military healthcare (National Endowments for the Arts, n.d.), and is established at 11 military facilities throughout the United States (Bronson et al., 2018). Before considering performance in clinical work, music therapists address functional rehabilitation (e.g., cognition, speech/language, sensorimotor) and behavioral health needs (Bronson et al., 2018). For example, a United States Army Captain co-authored a case report citing music therapy co-treatment as integral to his interdisciplinary rehabilitation. It motivated him to surpass his functional and psychological goals, ultimately leading to performances on local, national, and international platforms (Vaudreuil et al., 2018).

### Performance as a Vehicle for Rehabilitation and Social Transformation

**FIGURE 1:**
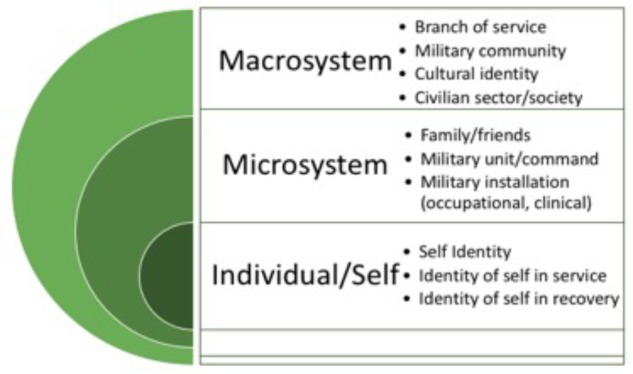
Systems of social transformation for service members (based on Bronfenbrenner, 1977).

Performance contributes to service members’ agency in recovery processes and has the potential to facilitate change and enhance overall perception of self and others by mastering challenges of TBI and PTSD. We find it helpful to conceptualize the levels of transformation per Bronfenbrenner’s Ecological Systems theory (Bronfenbrenner, 1977), specifically how people interact, adapt, and develop within their environments. Social transformation is a fluid process for the individual and occurs within the microsystem (e.g., installation, clinic) and in the macrosystem (e.g., culture, community). Figure 1 shows a model adapted from Bronfenbrenner to reflect the levels of environment that impact military personnel.

Performance provides opportunities for service members to be viewed in ways that enhance interactions within different environments (e.g., family, occupational, and community). It allows service members to increase awareness of self-perceptions, challenge belief systems, and view treatment as an evolving mechanism for change. Historically, the image of the warrior across many cultures has represented strength, power, and indestructibility, while demonstrating emotions and vulnerability has been interpreted as weakness (Peace et al., 2016). Performance allows reframing of the service members’ and audiences’ perspectives, transforming vulnerability to be recognized as resilience. A major transformation that service members ascribe to performance is a shift in perception from primarily internal to one that supports simultaneous awareness of internal and external processes. They describe public performances as validating and report that it allows expression of their inner-selves while feeling connected to audiences.

Through observing service members’ performances, audiences, commonly comprised of family, peers, clinicians, and community members can: (1) increase awareness of service members’ unique strengths by observing their products of music therapy, (2) comprehend service members’ experiences in an innovative way, (3) shift perspective of service members as healthy and resilient, rather than viewing deficits, and (4) develop an understanding of how to construct supportive communities that better accommodate service member (re)integration.

### Clinical Considerations of Performance in Music Therapy

Performance is not a requirement of music therapy, rather an optional component that can serve to cultivate tactical creativity and culminate therapeutic experiences. If service members decide to perform, they have complete autonomy over their experience including: (1) target audience, (2) arrangement/dynamics, (3) accompaniment/instrumentation, and (4) co-performance participation. Co-performances can occur between service member and peer(s), music therapists, providers from other disciplines, visiting musicians, or any combination of these. In some cases, performances are recorded and used as auditory feedback for service members to gain insight, further process experiences, and have as a tangible item to take with them.

It is important to consider how music therapists utilize the therapeutic relationship to support performance. Transparency regarding potential benefits and risks of performance must be addressed prior to client engagement in performance. Service members may be hesitant about performing personal content and therapists must remain acutely aware of this by placing emphasis on preparation, continuous evaluation of the experience, and offering emotional support throughout (Baker, 2013). There are many reasons why service members may feel uncomfortable sharing their experiences through performance, including stigma of injury and the perceived lack of understanding from the civilian sector. If the service member does not possess the internal resources to withstand increased vulnerability, performance can be counter-therapeutic or re-traumatizing; thus, the clinician must recognize the client’s unique capacity for risk-taking (Baker, 2013). In addition, it is essential that informed consent is established prior to the client engaging in performance. This provides consistency of client autonomy and optimization of choice and control when introducing integration of performance in music therapy. Ethical safeguards were employed with the service members highlighted in the case report section of this article to mitigate risk of performance. Clear and continuous communication on a spectrum from intervention to performance was incorporated throughout pre-performance preparation, support during the performance, and post-performance processing.

## Performance Initiatives and Case Examples

We present here two examples of performance initiatives integrated into military music therapy programs followed by two brief case reports of service members who received music therapy in intensive outpatient and longitudinal care at the NICoE and performed at the Creative Arts Café as part of their treatment. Case examples are based on clinical observations, service member interviews, and focus on how performance shaped social transformation. Both service members were interviewed about their experiences several months after treatment. A content analysis of the interviews focused on three areas: (1) music therapy treatment, (2) transformation through performance, and (3) post-music therapy transition. Please note that pseudonyms are used to maintain client confidentiality.

### Resounding Joy’s Semper Sound Band

The Semper Sound Band (est. 2011) is a component of Resounding Joy’s Military Music Therapy program (Resounding Joy, n.d.). Performance was a natural progression for service members who first received individual music therapy and progressed to group sessions. Mutual interest amongst group members led to a ‘jam group,’ which resulted in the formation of the band. Performance allows band members to showcase talents to family members, peers, clinicians, and communities through events, on and off base.

### Creative Forces Creative Arts Café

Based on the concept of the Semper Sound Band, the Creative Arts Café is an initiative developed at the National Intrepid Center of Excellence (NICoE) in 2016 and replicated across Creative Forces sites (Bronson et al., 2018). The Creative Arts Café is a performance space where service members can highlight certain aspects of their treatment processes in creative arts therapies. It serves as a platform for service members, family members, and staff to share creativity through the arts. It supports the clinic to community continuum, as service members often first perform in music therapy and then may decide to perform on base and in the greater community. It is important to note that the Creative Arts Café initiative is open to all forms of artistry (music, art, dance, writing/spoken word, theatre, etc…), however, the case reports presented below focus exclusively on music performance.

### Case Report 1

#### Background

“Greg” is a male in his twenties and was a Sergeant (SGT) in the United States Army. He was an unrestrained passenger in a Humvee rollover in 2014. He suffered intracranial hemorrhages, presented unresponsive, and was intubated. Greg was medevacked, stabilized, and returned to the United States for ongoing treatment. He was referred to music therapy by a nurse case manager for cognitive deficits due to severe TBI. Greg received a music therapy assessment in January 2016 and received weekly individual and small group sessions for 15-months as part of outpatient treatment.

#### Music Therapy Treatment

Upon initial music therapy assessment, it was determined that Greg was experiencing expressive speech issues in addition to memory and coordination deficits. The music therapist consulted with speech therapy to create integrative treatment approaches. Music therapy sessions were designed to assist his breathing, speech fluency, and rhythmic phrasing. A few months into music therapy, Greg and a fellow service member were working on a song that supported shared speech and cognition goals. The music therapist determined that it was appropriate to offer performance as an opportunity to: (1) encourage active participation in treatment, (2) enhance self-practice, and (3) engage with an audience while applying skills learned in music therapy. Greg and his peer were working on the song, “We Didn’t Start the Fire” by Billy Joel and opted to perform it as a duet. In his interview “Greg” stated, *“I chose that song, because I knew for a fact that it would help with my speech...and it helped with my memory.”*

Therapeutic singing, rhythmic cueing, and vocal prosody exercises were used to address hypernasality, slurred speech, fluidity, intonation, and articulation. Greg stated, *“[Music therapy] helped my pronunciation of words, [performance] pushed me harder in music therapy, which in turn pushed my brain harder to focus on my words and my motor functions.”* He furthermore acknowledged how performance had helped him address vocal and cognitive changes experienced since his accident: *“My voice changed since I got hurt...I didn’t really want to talk to people... With music therapy, I learned not to be ashamed of my voice, and that it won’t get better if I don’t talk. It helped me with my memory because I had to remember lyrics, chord changes, and everything else...it made a great impact overall.”* Through performance in music therapy, Greg gained insight into personal struggles and was motivated to continue engaging in independent practice and performance. He shared, *“Watching expressions of people I don’t even know...seeing and feeling that someone you’ve never met before [is] showing happiness and joy from your performance…that is what is really empowering.”*

#### Performance as Motivation for Therapeutic Work

Greg participated in monthly performances as a therapeutic tool to provide awareness of his successes in rehabilitation goals. Eventually, he was able to simultaneously sing and play, which is a high-level cognitive process and an ongoing goal. He recounts, “*Between music therapy sessions, I was rehearsing. Every time I got stuck on a word, I went back to my room and [would] say it, and my speech got clearer. It made me feel amazing after [performing] because, honestly I noticed every time I did something, people would be amazed.”* Performance supported Greg in changing from an internal focus to being able to communicate with others using music. This was observed by a shift in song selections from those that solely challenged his speaking/singing ability to songs that he connected with on a deeper level. Intentional song selection eventually led to Greg writing original compositions that reflected his belief systems and included messages to inspire himself and others. He spoke about the emotional benefits and comfort that he experienced from performance in music therapy, *“With music, that’s your friend, your therapist, and everything beyond, and it doesn’t have anything bad to say about you ever.”*

#### Generalization of Music Therapy in Transition

When Greg started music therapy, it was difficult and uncomfortable for him to speak on command or initiate conversations. Through the process of preparing for performances, which also included verbal song introductions, he continued to make clinical gains post-music therapy treatment, including speaking at his retirement ceremony and national public performances. Greg medically retired in May 2017 and is currently attending college with aspirations to become a motivational speaker. He reflected about his experiences performing in music therapy, “*[Performance] worked my body harder. If I did not have music therapy, I would not be doing as well as I am today. I [still] incorporate what music expresses in my life.”*

### Case Report 2

#### Background

“Chad” is a male in his thirties and was a Staff Sergeant (SSgt) in the United States Marine Corps. He served multiple combat deployments over his 13 years of service, during which he experienced blast exposure. Upon homecoming, Chad was diagnosed with mild TBI, chronic PTSD, and other combat-related psychological health concerns. He initially engaged in individual and group music therapy through the intensive outpatient program. Chad continued music therapy for 1 year post-IOP, in weekly individual sessions.

#### “Tuning in” to Treatment

Chad’s music therapy goals addressed pervasive symptoms of chronic PTSD, as well as mild TBI issues of cognition, emotional regulation, and expression. Music therapy sessions incorporated various interactive experiences (e.g., active music making, lyric analysis, songwriting) to assist him in processing his military service and positively reframing self-perception and identity. Chad often selected songs by preferred artists to express things that were difficult to communicate prior to music therapy. He recounts, “*Something about music made it easier to talk about issues that were bothering me. We spoke about issues that I hadn’t even discussed with my [psycho]therapist at that point*.” After 8 months of individual sessions, Chad opted to perform a song at a Creative Arts Café that he had been preparing for his wedding. The integration of personally meaningful music into music therapy motivated his engagement in performance. Lyric analysis and songwriting facilitated emotional expression, as Chad modified songs to reflect his values. He shared, “*I was able to add a spin on the song from my perspective, with help from the music therapist, which involved changing lyrics at the end,* “*Mercy,” look what’s become of us/One by one we turned it around/Maybe carry on just a little bit longer/God’s gonna give us what we need”* (adapted from “Mercy” Dave Matthews Band, 2012).

#### Transforming Trauma

A song of particular significance that Chad subsequently performed was an arrangement of “Walls” by Kings of Leon. He reflected on the song’s symbolism,*” ‘When the walls come down,’ that’s how I felt music therapy was helping me...my walls coming down.*” Chad’s musical engagement often inspired feelings of hope and reduction of personal guilt as reflected in his comment, *“I feel like singing or even whistling means that there is some kind of happiness inside me, and it’s not all dark...Music therapy is like God answering my prayers. A light in the darkness, and a reassurance that while I have experienced horror, I am not the cause of it all.”* He shared how performing helped him reduce isolation, *“Performances can help to reconnect with people and even give others a chance to understand me more.”* The reciprocal experience of self-understanding while feeling “heard” by the audience allowed for continuous progression in treatment.

#### Moving to the Next “Stage”

As Chad began preparing for his transition from active duty, he transferred skills developed through performance to continue growing in his life and relationships. His evolving interpretations of songs he performed in music therapy reflected his progression in treatment. For example, when he first used “Mercy” in sessions, the meaning was focused on in-the-moment needs, specifically symptom management. It progressed to address broader areas, namely humanization and becoming a better partner. Ultimately, Chad sang and played bass and percussion in a multimedia project of “Mercy” that he presented this to his wife, family, and friends at his wedding. He speaks about how the spirituality of the song aligned with his transformation in recovery, “*The first line in the song is, “Don’t give up”. Even though I felt like I had so many times, I didn’t give up, and to this day I am alive and not giving up. It was a sad song for me when the demons were in control, but God transformed the song into healing when it took on a new meaning for my wife and I, when we listened to it together. It transformed again as we grew as a couple, and we both went through really hard times along the path to recovery. [My wife] stayed with me through everything...there was no way I could let this song not be a part of our wedding day and music therapy was the reason it became a part of it.”* Chad medically retired in July 2017.

## Conclusion

Performance is an impactful tool for music therapists to utilize in support of clients’ transformative processes. When integrated with apt intention and appropriate oversight, performance can be clinically conducive, informative, and reciprocally beneficial for service members and audiences. Performance helps educate military leadership, clinical providers, and the general public about the power of incorporating creative expression into structured environments. Creative Arts Cafés are models replicated across multiple sites that serve to bridge the clinic to community, supporting the overall transformation of service members and their families by validating their experiences and allowing for expression of their inner-selves while feeling connected to audiences. Musical performance benefits our society as a whole, supporting personal, collective, and societal transformation. This shapes the performer as well the perception of audiences and builds stronger, healthier communities in which individuals thrive.

## Disclosure Statement

The views expressed in this article are those of the author and do not reflect the official policy of the Department of the Army/Navy/Air Force, Department of Defense, or United States Government. The identification of specific products, scientific instrumentation, organizations, individuals or compositions is considered an integral part of the research endeavor and does not constitute endorsement or implied endorsement on the part of the author, DoD, or any component agency.

## Ethical Disclosure

The case studies presented in this article were determined exempt by the NICoE/WRNMMC institutional review committee. Written informed consent was obtained from the participants for the publication of the case reports.

## Ethics Statement

This study was carried out in accordance with the recommendations of the National Intrepid Center of Excellence/Walter Reed National Military Medical Center PAO and designated as exempt from ethics committee review. All subjects gave written informed consent in accordance with the Declaration of Helsinki. The protocol was approved by the National Intrepid Center of Excellence/Walter Reed National Military Medical Center PAO.

## Author Contributions

RV is a board-certified music therapist and served as contract support to the NICoE through National Endowment for the Arts, Creative Forces: NEA Military Healing Arts Network. During her tenure at the NICoE, she was the primary music therapist of the service members who were highlighted in the case report section of this article. As lead author, she conceptualized this article and took the lead in writing the article. She was responsible for final edits of the manuscript. HB contributed to content analysis of the interviews and writing the manuscript. JB contributed to sections of the manuscript.

## Conflict of Interest Statement

The authors declare that the research was conducted in the absence of any commercial or financial relationships that could be construed as a potential conflict of interest.
